# Application of Artificial Intelligence in Ophthalmology: An Updated Comprehensive Review

**DOI:** 10.18502/jovr.v19i3.15893

**Published:** 2024-09-16

**Authors:** Hesam Hashemian, Tunde Peto, Renato Ambrósio Jr, Imre Lengyel, Rahele Kafieh, Ahmed Muhammed Noori, Masoud Khorrami-Nejad

**Affiliations:** ^1^Translational Ophthalmology Research Center, Farabi Eye Hospital, Tehran University of Medical Sciences, Tehran, Iran; ^2^School of Medicine, Dentistry and Biomedical Sciences, Centre for Public Health, Queen’s University Belfast, Northern Ireland, UK; ^3^Department of Ophthalmology, Federal University the State of Rio de Janeiro (UNIRIO), Brazil; ^4^Department of Ophthalmology, Federal University of São Paulo, São Paulo, Brazil; ^5^Brazilian Study Group of Artificial Intelligence and Corneal Analysis – BrAIN, Rio de Janeiro & Maceió, Brazil; ^6^Rio Vision Hospital, Rio de Janeiro, Brazil; ^7^Instituto de Olhos Renato Ambrósio, Rio de Janeiro, Brazil; ^8^School of Medicine, Dentistry and Biomedical Sciences, Queen’s University Belfast, Northern Ireland; ^9^Department of Engineering, Durham University, United Kingdom; ^10^School of Rehabilitation, Tehran University of Medical Sciences, Tehran, Iran; ^11^Department of Optical Techniques, Al-Mustaqbal University College, Hillah, Babylon 51001, Iraq

**Keywords:** Artificial Intelligence, Ophthalmology, Prognosis, Screening, Treatment

## Abstract

Artificial intelligence (AI) holds immense promise for transforming ophthalmic care through automated screening, precision diagnostics, and optimized treatment planning. This paper reviews recent advances and challenges in applying AI techniques such as machine learning and deep learning to major eye diseases. In diabetic retinopathy, AI algorithms analyze retinal images to accurately identify lesions, which helps clinicians in ophthalmology practice. Systems like IDx-DR (IDx Technologies Inc, USA) are FDA-approved for autonomous detection of referable diabetic retinopathy. For glaucoma, deep learning models assess optic nerve head morphology in fundus photographs to detect damage. In age-related macular degeneration, AI can quantify drusen and diagnose disease severity from both color fundus and optical coherence tomography images. AI has also been used in screening for retinopathy of prematurity, keratoconus, and dry eye disease. Beyond screening, AI can aid treatment decisions by forecasting disease progression and anti-VEGF response. However, potential limitations such as the quality and diversity of training data, lack of rigorous clinical validation, and challenges in regulatory approval and clinician trust must be addressed for the widespread adoption of AI. Two other significant hurdles include the integration of AI into existing clinical workflows and ensuring transparency in AI decision-making processes. With continued research to address these limitations, AI promises to enable earlier diagnosis, optimized resource allocation, personalized treatment, and improved patient outcomes. Besides, synergistic human-AI systems could set a new standard for evidence-based, precise ophthalmic care.

##  Introduction

Artificial intelligence (AI) has shown a tremendous potential across many fields, including health care.^[1]^ In ophthalmology, AI applications are up-and-coming due to the wealth of digital imaging data and objective metrics.^[2,3]^ Fundus photography, optical coherence tomography (OCT), and visual field testing provide large datasets suitable for developing machine learning algorithms to diagnose and manage eye diseases.^[4,5,6]^

The strategic integration of AI in ophthalmology has opened new possibilities for holistic ophthalmic clinical services.^[6,7]^ As global life expectancy rises, age-related eye diseases increase, further straining healthcare systems that currently struggle to meet new demands.^[8]^ AI can help identify patients with preventable vision loss earlier and optimize the allocation of medical resources through accurate predictions and personalized interventions.^[9]^

Early successes in medical AI have spurred considerable research into ophthalmic applications. In 2018, the IDx-DR became the first FDA-approved autonomous AI diagnostic system for diabetic retinopathy (DR) detection, marking a landmark achievement.^[10]^ Since 2008 or earlier, AI has been applied to diverse ophthalmology contexts, including screening and diagnosis of conditions such as DR, glaucoma, retinopathy of prematurity (ROP), and keratoconus.^[5,11,12,13,14,15]^ AI has also shown promise in treatment planning, predicting outcomes of interventions, and improving the efficiency of clinical workflows.^[16]^

However, translating AI research into viable clinical tools remains challenging. Algorithm performance depends heavily on the quality and diversity of training data. “Black box” AI systems that lack interpretability raise justified skepticism, and their seamless integration into existing clinical practice is yet to be achieved. Nevertheless, AI holds immense potential to provide clinicians with data-driven, objective assessments that could radically transform ophthalmic care.^[17]^ This review summarizes recent advances and challenges in applying AI to major domains in ophthalmology.

### Imaging Enhancement and Analysis

Deep learning (DL) has enabled transformative advances in ophthalmic imaging analysis. Regarding OCT, AI algorithms have been developed for image quality improvement, semantic segmentation, and extraction of clinically meaningful biomarkers.

Several groups of researchers have shown DL can effectively reduce OCT image noise and artifacts. Ouyang et al trained a generative adversarial network that significantly enhanced image quality rated by clinicians.^[18]^ Additionally, Noise2Noise algorithms leverage intrinsic redundancy in repeated scans to denoise images.^[19]^

Automated segmentation of retinal layers and quantification of morphological features like fluid and drusen are now feasible with AI. Schmidt et al engineered a DL algorithm focused on quantifying intraretinal cyst fluid that correlated robustly with manual grading assessments.^[20]^ Research has also indicated that AI-driven segmentation of choroidal neovascularization lesions aligns closely with clinician assessments in OCT angiography

(OCTA).^[21,23]^DL networks can also synthesize OCT data to estimate other modalities. Wang et al employed a cross-modality synthesis approach to produce OCTA images from structural OCT scans.^[24]^ The application of DL to visual field assessments, ultrasonography, and additional ophthalmic imaging techniques shows potential for measuring informative biological markers.

##  Diagnosis and Prognosis

AI has shown promising results in assisting the diagnosis and prognosis of various ophthalmic diseases. Several studies have developed DL algorithms using retinal fundus images to accurately detect referable DR. For example, De Fauw et al created a DL system that could diagnose referable DR and diabetic macular edema (DME) from OCT scans with sensitivity and specificity comparable to retina specialists.^[4]^

Burlina et al developed a DL algorithm, using over 130,000 color fundus photographs, that achieved a diagnostic accuracy of 91.6% for detecting moderate to advanced age-related macular degeneration (AMD).^[25]^ Grassmann et al trained a DL algorithm on 120,656 fundus images that could distinguish early from late AMD with 94.3% accuracy.^[26]^

In the context of glaucoma, several studies have applied DL to optic disc photographs for detecting glaucoma with high sensitivity and specificity.^[12,27]^ Asaoka et al developed a DL algorithm using OCT images.^[28]^ They reported that utilizing the deep feed-forward neural network classifier resulted in a markedly high area under the curve (AUC) of 0.926, with a confidence interval (CI) of 95% ranging from 89.8% to 95.4%. The efficacy of this model exceeded that of alternative machine learning methods such as the random forest (RF) algorithm, which yielded an AUC of 0.79 with a 95% CI ranging from 73.5% to 84.5%. Moreover, the gradient boosting method demonstrated an AUC of 0.776, with a 95% CI extending from 71.7% to 83.5%. Machine learning has also been applied to visual field data for detecting early glaucoma and predicting future visual field loss.^[29,30,31]^

The application of AI in the diagnosis and follow-up treatment of ROP has shown significant promise. The new AI algorithms, particularly DL models, analyze retinal images and identify signs of ROP with high accuracy. For instance, Brown et al demonstrated a DL system, trained on over 5000 wide-field retinal images, that achieved 93% sensitivity and 94% specificity for ROP.^[13]^ Additionally, Redd et al developed an AI platform for ROP screening that achieved an AUC of 0.96 for detecting type 1 ROP, indicating high diagnostic accuracy.^[32]^ These AI systems can assist clinicians by providing rapid and reliable assessments, thereby facilitating timely intervention and reducing the risk of vision loss. AI can also be used in the follow-up treatment of ROP to monitor disease progression and predict outcomes, enabling personalized treatment plans. Screening for ROP faces different challenges, particularly in rural areas due to a shortage of specialists and services. Telemedicine has emerged as a viable alternative to clinical examinations in areas lacking optimal screening conditions, and tele-education programs aims to enhance physician training and optimize ROP care.^[33]^ By integrating AI into clinical workflows, healthcare providers can enhance the efficiency and effectiveness of ROP management, ultimately improving patient outcomes.

In short, numerous studies have shown the AI potential to assist in screening, diagnosis, prognosis, and referral recommendations for major blinding eye diseases. With further validation, AI can be clinically applied to improve patient outcomes through early diagnosis and personalized management.

### Application of AI Models and Algorithms in Anterior Segment Diseases

#### Keratoconus 

Keratoconus is a bilateral non-inflammatory corneal ectasia characterized by corneal thinning and irregular astigmatism.^[34]^ Early diagnosis of keratoconus allows for identifying individuals who are more likely to develop progressive ectasia. The diagnosis of keratoconus is crucial for preventing disease progression and visual loss.^[35]^ However, subtle changes in early keratoconus can be challenging to be detected by examination alone. Advanced imaging techniques such as corneal topography, tomography, and biomechanics^[36,37]^ provide objective data that can aid diagnosis, but their interpretation requires expertise. This is where AI can assist by detecting patterns not discernible to the human eye.

Many researchers have utilized AI-based techniques such as artificial neural network (ANN), support vector machine (SVM), and random forest (RF) algorithms to examine topographical and tomographical datasets in the context of keratoconus detection.^[38,39]^ Arbelaez et al formulated an SVM model predicated on Scheimpflug tomography data elements, which encompassed pachymetry maps, keratometry, and higher-order aberrations; the model achieved a 95% accuracy rate in differentiating between normal and keratoconic corneas.^[40]^ Smolek et al assessed an ANN model against conventional topographic indices used for keratoconus classification, and they noted comparable sensitivity but enhanced specificity.^[41]^ In more recent developments, sophisticated DL models, particularly those based on convolutional neural network (CNN) architectures, have been reported to deliver superior performance. Lavric et al developed a CNN model called KeratoDetect that uses raw topography data and could achieve 98.9% accuracy in detecting keratoconus.^[42]^

Apart from diagnosing established keratoconus, detecting subclinical or early keratoconus is also essential to prevent ectasia after refractive surgery. Lopes et al introduced the Pentacam random forest index (PRFI), which yields 85.2% sensitivity and 96.6% specificity for subclinical keratoconus.^[43]^ AI models have also been applied to analyze data from corneal biomechanics devices such as the ocular response analyzer (ORA) and Corvis ST.^[43,44]^

The combined tomographic and biomechanical parameter has been more effective than either method used separately.^[45]^ Ambrosio et al assessed tomographic-biomechanical index (TBIv1) using Pentacam and Corvis.^[46]^ In patients with bilateral keratoconus and those with very asymmetric ectasia, the TBIv1 algorithm demonstrated high accuracy in detecting ectasia, with an AUC of 0.999, sensitivity of 98.5%, and specificity of 98.6% using a cutoff value of 0.5. Similarly, the TBIv2 algorithm—a novel RF model incorporating 18 features in 156 trees and developed through 10-fold cross-validation—showed a comparable AUC of 0.999 for diagnosing clinical ectasia. The TBIv2 algorithm exhibited a sensitivity of 98.7%, specificity of 99.2%, and cutoff value of 0.8, with no significant difference compared to TBIv1 in diagnosing clinical ectasia (DeLong, *P* = 0.818). Nevertheless, for the diverse group of cases with normal topography from patients with very asymmetric ectasia (VAE-NT), TBIv1 had an AUC of 0.899 (76% sensitivity and 89.1% specificity [cutoff: 0.29]), while TBIv2 had a significantly higher AUC of 0.945 (DeLong, *P* < 0.0001), with 84.4% sensitivity and 90.1% specificity (cutoff: 0.43).

The published studies demonstrate the potential of AI in upgrading the detection of both established and subclinical keratoconus. However, the heterogeneity between current studies highlights the need for a standardized methodology and widely validated datasets. As more advanced DL models are developed and tested prospectively on diverse populations, AI could become an indispensable tool for early diagnosis of keratoconus in clinical practice.

#### Dry eye disease (DED)

Dry eye disease (DED) is a highly prevalent multifactorial condition of the tear film and ocular surface. The core mechanisms are aqueous tear deficiency and excessive evaporation, which lead to damage and inflammation.^[47,48]^ Population-based studies estimate the global prevalence of DED symptoms to be between 5% and 50%, with higher rates among women and older people.^[49,50]^ DED can also significantly reduce quality of life and visual functioning.^[51,52]^

The diagnostic challenges in this context arise from the discordance between signs and symptoms, lack of consensus on definitive criteria, and variability in clinician diagnosis.^[53,54]^ The Dry Eye Workshop II (DEWS II) recommends a multi-pronged approach combining symptom questionnaires, tear film tests, and ocular surface staining.^[55]^ However, specific thresholds are still debated. For example, different studies show that the cutoff point for mild DED ranges from 12 to 22 points, according to the ocular surface disease index (OSDI) questionnaire.^[56,57]^ Tear osmolarity cutoffs also range from 308 to 316 mOsm/L.^[58,59]^ Given this inconsistency, it is challenging to apply AI techniques that rely on definitively labeled training data. Nevertheless, several research groups have recently published promising works on AI-assisted diagnosis of DED. In a study by Siyan et al, 82,236 meibography images from 20,559 subjects were processed for classification using the SimCLR neural network. Image segmentation was conducted using the UNet model to identify meibomian gland areas, and clinical evaluations such as tear breakup time, tear meniscus height, and glandular atrophy were carried out on a subset of 280 individuals.^[60]^ The findings confirmed that the SimCLR neural network effectively sorted patients with dry eye into six unique image-based groups. These groups exhibited noteworthy differences in tear film integrity and meniscus height, with some patients showing significant meibomian gland atrophy, varied degrees of corneal staining, and differences in gland size. The identified subtypes also corresponded to variations in meibum quality and the meibomian glands' structural characteristics. In another study, Wang et al drew on AI to investigate meibomian gland morphology.^[61]^ A collection of 1443 meibography images were annotated and split into training (1039 images) and testing (404 images) sets. These sets were used to develop and validate DL models for gland segmentation and ghost gland detection. The models achieved a 63% mean intersection over union for segmentation, 84.4% sensitivity, and 71.7% specificity for ghost gland identification, and low local contrast was reported as a key predictor for ghost glands.

OCT is another modality in recent AI research. Anterior segment OCT (AS-OCT) captures objective, high-resolution images of the ocular surface and tear film. Chase et al discovered that an independent DL algorithm showcased an accuracy of 84.62%, a sensitivity of 86.36%, and a specificity of 82.35% in pinpointing DED by evaluating tear meniscus height and epithelial abnormalities in AS-OCT images.^[62]^ While there is a need to conduct further extensive clinical validation, this research underscores the prospective benefits of incorporating AI into diagnostic imaging processes for DED.

The aforementioned applications of AI are promising but remain in the early stage. Comprehensive multicenter studies are needed to train more powerful AI models that can be incorporated into clinical practice. Defining DED subtypes based on multimodal biomarkers as reference standards could strengthen diagnostic accuracy. Advances in explainable AI could also help determine the key features used by models and improve AI interpretability before clinical translation. Overall, AI and machine learning techniques show immense capability for enhancing DED management if major existing challenges around validation and interpretation can be overcome.

#### Refractive surgery

Elective refractive surgery aims to enhance patient's quality of life and satisfaction by reducing the dependence on glasses or contact lenses through reshaping the cornea with laser ablation (e.g., PRK and LASIK procedures) or intraocular lens (IOL) implantation. However, there are risks like dry eye, IOL miscalculations, and iatrogenic ectasia.^[63]^ AI techniques are being developed to improve outcomes in preoperative screening, surgical planning, and postoperative monitoring.^[64,65]^

Preoperative screening before corneal refractive surgery (CRS) is vital to prevent postoperative ectasia. Arbelaez et al evaluated multiple AI models, including SVM, RF, and ANN, for detecting eyes at risk of ectasia based on topography and tomography data.^[40]^ Among these three models, SVM performed best with an AUC of 0.97 and 94% sensitivity. Xie et al developed a DL system called PIRSS using Pentacam data that distinguished keratoconic eyes from normal eyes with 95% accuracy.^[66]^ Such AI tools can enhance risk stratification before CRS.

#### IOL power calculation

Accurate preoperative measurement of ocular parameters is crucial for appropriate IOL power selection and optimization of visual outcomes after cataract surgery.^[67,68]^ However, errors can occur due to variations in measurement techniques and devices. AI has been applied in developing integrated systems that take into account multiple ocular variables to recommend the ideal IOL power for a given patient.^[69,70]^ Some studies have recommended the ideal IOL power based on preoperative data.^[67,72,73]^ Traditional regression formulas have limitations in IOL power calculation for post-refractive and short eyes. Newer methods such as Barrett Universal II use machine learning to optimize IOL constants but may still have errors.^[71]^ More advanced AI models that can account for multiple ocular parameters are being developed to improve the predictability of target refraction.

### Screening

#### Diabetic retinopathy (DR)

DR is a leading cause of vision loss and preventable blindness globally. Approximately one-third of patients with diabetes develop DR.^[74]^ The timely identification and intervention for DR are imperative to avert permanent visual impairment.^[75]^ Nevertheless, the manual examination of retinal images for DR detection is a labor-intensive and costly process that necessitates the expertise of trained professionals. These factors have constrained the extensive adoption of DR screening initiatives.^[76]^

Recent advances in AI, especially DL, provide new opportunities for automated analysis of retinal images for DR screening.^[4]^ Several research groups have developed and validated DL algorithms to detect DR using color fundus photographs.^[77,78,79]^ A pivotal study by Gulshan et al demonstrated that a DL algorithm could identify referable DR (moderate NPDR or worse) with 90% to 98% sensitivity and specificity on two large independent test datasets.^[80]^ The algorithm performed on par with expert human graders.^[10,81]^ Accordingly, the first autonomous AI diagnostic system, IDx-DR (IDx Technologies Inc, USA), received FDA approval in 2018 for DR screening.^[10]^ Another automated DR screening system is AEYE-DS, which is the third AI system approved by the FDA for screening DR from retinal images. The FDA approval data indicate that AEYE-DS, based on two macula-centered images per patient, has a sensitivity of 92.98% and a specificity of 91.36% for more than mild DR.^[82]^

Many DL models employing diverse CNN architectures have demonstrated impressive efficacy in identifying referable DR, commonly achieving an AUC within the range of 0.94 to 0.99.^[77,78,79]^ Li et al devised a composite DL algorithm that was trained on fundus photographs sourced from various ethnic groups.^[83]^ Upon evaluation using internal validation datasets, this algorithm attained a receiver operating characteristic curve (ROC AUC) score of 0.989, sensitivity of 97.0%, and specificity of 91.4% in the detection of referable DR. Further assessment on an independent, diverse dataset also demonstrated the good performance of the proposed algorithm, indicating an ROC AUC of 0.955, 92.5% sensitivity, and 98.5% specificity. Focusing on the analysis of the fovea and optic disc areas, Ramachandran et al introduced a multi-tiered DL framework that replicates the clinical evaluation process and has an AUC of 0.98.^[84]^ Diagnostic accuracy of this model is enhanced through the incorporation of multimodal retinal imaging techniques. For example, compared to either modality alone, combining color fundus photos and OCT imaging improves sensitivity for identifying referable DR.^[81]^

AI-based DR screening systems can provide automated, quick, and accurate screening at low costs. Real-world validation studies have shown that these systems can be effectively implemented in clinical settings.^[85,86]^ The IDx-DR device was validated in 10 primary care sites, demonstrating a sensitivity of 87.2% and specificity of 90.7% for more-than-mild DR.^[10]^ An AI system developed by Rajalakshmi et al was implemented in 181 vision centers across India, and it screened over 150,000 patients in real time.^[85]^ The AI software showed 95.8% sensitivity and 80.2% specificity for DR.

However, some key challenges need to be addressed before the widespread adoption of AI-based DR screening.^[4,79]^ The performance of algorithms can vary significantly depending on the camera used and image quality. Most current models are tested on high-quality retinal images but may not perform as well on low-cost fundus cameras or mobile phone-based images.^[85]^ Extensive training with diverse images is required to make the algorithms more robust. The datasets used for training algorithms also need to include broader population groups in terms of age, ethnicity, and risk factors.^[4]^ Regulatory approval and medicolegal implications of AI screening must be established.^[10]^ Finally, effective integration of AI into existing screening workflows requires careful planning for infrastructure, quality control, coordination, and personnel training.^[86]^

If these challenges are appropriately addressed, AI-assisted and autonomous DR screening will facilitate the creation of cost-effective programs that can achieve widespread coverage and especially benefit underserved populations.^[79,80,83]^ This would lead to early detection and treatment, ultimately preserving vision and preventing blindness for millions of patients with diabetes worldwide.

#### Glaucoma 

Glaucoma is a leading cause of irreversible blindness worldwide,^[87]^ and its early diagnosis is key to initiating treatment and preventing vision loss.^[88]^ However, population-based glaucoma screening is challenging due to the asymptomatic nature of early stage of the disease and the requirement to run multiple diagnostic tests by trained experts.^[89]^

Recent research has explored the application of DL algorithms to diagnose glaucoma by analyzing optic nerve head morphology in retinal fundus photographs. Li et al developed a DL model using 48,116 fundus images, and it could distinguish patients with glaucoma from healthy individuals with an AUC of 0.986, sensitivity of 95.6%, and specificity of 92.0%.^[12]^

Several DL frameworks have combined fundus image analysis with clinical risk factors to improve the accuracy of glaucoma screening. Diaz-Pinto et al developed an AI-assisted tool that integrated fundus image data with age and family history, achieving an AUC of 0.9605, sensitivity of 0.9346, and specificity of 0.8580 for discriminating between healthy and glaucoma eyes.^[90]^ Arbabshirani et al combined fundus image features from a DL model with OCT and visual field data, and the results improved sensitivity and specificity for glaucoma diagnosis compared to single modalities.^[91]^

There are certain challenges for glaucoma screening based on fundus photography and using AI. The appearance of the optic disc can vary significantly depending on factors such as refractive error, media opacity, and acquisition device.^[92]^ Low-quality images with poor illumination, low contrast, or motion artifacts reduce the accuracy of algorithms,^[12]^ and diagnostic performance is significantly affected in cases with tilted disc configuration.^[93]^ Additionally, extensive training with diverse fundus images is essential to promote the robustness of DL algorithms.^[94]^

Sensitivity is lower for identifying early glaucoma, when disc changes may be subtle.^[95]^ Screening accuracy of early glaucoma could be improved by combining optic disc morphology analysis with assessment of the retinal nerve fiber layer and ganglion cell complex on OCT scans.^[12,96]^ Multimodal AI frameworks incorporating fundus, OCT, and visual fields will most likely provide robust performance in real-world screening.^[97]^ Finally, prospective validation in targeted screening populations will be vital before widespread clinical implementation.^[98]^

In summary, DL-based automated analysis of optic disc morphology in fundus photographs shows promise as a tool for community-based glaucoma screening. With continuous improvements in algorithms and their integration with other clinical data, fundus image-based AI screening can potentially enable cost-effective programs to detect glaucoma early and preserve patients' vision.

#### Age-related macular degeneration (AMD)

AMD is a leading cause of vision impairment and blindness among the elderly worldwide. The prevalence of AMD is projected to rise exponentially with aging populations.^[99]^ Early diagnosis and risk stratification of AMD is essential to optimize management and prevent vision loss.^[100]^ However, routine screening for AMD is challenging since it requires skilled graders and time-consuming manual analysis of retinal images.^[101]^

Recently, DL has emerged as a promising technique to automate the analysis of retinal color fundus photographs and OCT images for AMD screening and severity classification.^[102]^ Multiple research groups have developed DL algorithms that detect AMD with high accuracy comparable to clinical experts.^[25,26]^ Treder et al validated an algorithm to distinguish normal and AMD OCT scans.^[103]^ They reported that the model achieved 100% training accuracy and validation accuracy with a low cross-entropy loss of 0.005. When tested on an AMD dataset, the model produced high average anomaly detection score of 0.997 ± 0.003. On a healthy comparison group, it resulted in a significantly lower average score of 0.9203 ± 0.085 (*P* < 0.001). Schmidt-Erfurth et al showed an AI system could predict progression to late AMD and could offer high sensitivity and specificity for identifying new-onset choroidal neovascularization in patients with AMD.^[104]^

DL algorithms have also been applied for drusen quantification on fundus photographs,^[105]^ geographic atrophy measurement on OCT,^[106]^ and prediction of anti-VEGF treatment needs.^[107]^ De Fauw et al demonstrated that a DL model, with an accuracy matching experts could triage AMD cases into routine, urgent, or emergency categories based on OCT scans.^[4]^ Real-world utility of AI-assisted AMD screening has been confirmed through different studies, including a multicenter evaluation of an autonomous AI system for diagnosing AMD in primary care settings.^[10]^

However, it remains a challenge to generalize DL algorithms for AMD across different populations.^[12]^ Most current models are developed and tested in European ancestry cohorts, and they need to be evaluated in other ethnicities.^[108]^ Model performance also depends significantly on image quality, motion artifacts, media opacity, and segmentation errors, which are currently key challenges.^[109]^ Combining color fundus photos with other modalities such as OCT and fundus autofluorescence improves diagnostic accuracy.^[26]^ Finally, it is crucial to improve the prognostic abilities of algorithms to predict longitudinal outcomes.^[110]^

In summary, AI-based analysis of retinal images shows immense potential to automate population screening and improve early AMD detection and risk stratification. With careful validation across diverse cohorts and integration into screening workflows, AI could enable cost-effective programs that achieve widespread coverage and, ultimately, preserve the vision for patients with AMD.^[26]^ Obtaining such benefits requires collaboration between clinicians, data scientists, and policymakers to transition from proof-of-concept studies to clinical implementation.^[5]^

### Treatment 

While most AI applications have focused on screening and diagnosis, more recent studies explore using AI to support treatment decisions. For example, AI algorithms can use OCT biomarkers to predict response to anti-VEGF therapy in patients with neovascular AMD.^[111,112]^ Other algorithms can determine the need for anti-VEGF reinjection in patients with neovascular AMD and DME.^[113,114]^ Beyond anti-VEGF therapy, AI has been applied to plan for laser photocoagulation. The NAVILAS laser system uses fluorescein angiography registration and an AI model to target spots identified for laser treatment,^[115]^ resulting in improved precision and reduced treatment burden.

AI could optimize treatment regimens by forecasting disease progression and recommending patient-specific management plans.^[116,117]^ However, three are a number of challenges in realizing the potential of AI-based treatment planning. Most works in this area have relied on small sample sizes from single institutions, and multicenter trials are needed to demonstrate generalizability and clinical utility. The interpretability of AI model predictions also requires improvement. Nevertheless, AI represents a promising approach to optimize therapies, avoid undertreatment or overtreatment, and improve outcomes in major retinal diseases.

### Limitations and Challenges

While AI shows promise in enhancing optometry and ophthalmology, its limitations and challenges still need to be addressed. One fundamental limitation is the quality and representativeness of the datasets used to train AI algorithms.^[3,5]^ Low-quality images or biased datasets can result in poor model performance and lack of generalizability across diverse patient populations. There is a need for large, high-quality, and diverse training datasets that capture the breadth of pathology and demographics in the real world.^[118,119]^ The curation of datasets, however, is an intensive process requiring clinical expertise.

Another major challenge is regulatory approval and the medicolegal implications of AI systems.^[120,121]^ Most existing systems are still in the research phase and have not gone through rigorous clinical validation and regulatory clearance. Questions around legal responsibility in cases of misdiagnosis and harm must be resolved, and regulatory bodies are still developing appropriate evaluation frameworks for AI technologies.^[122]^

While black box AI approaches like DL have shown remarkable performance, they lack interpretability and transparency in making decisions.^[123]^ Having insight into the AI logic and representing uncertainty in outputs are essential for clinician trust and decision-making.^[124]^ In this regard, it is encouraging to note that researchers are actively exploring new methods to increase model interpretability.

Another barrier to real-world deployment of AI consists of adapting clinical workflows and software interfaces to seamlessly integrate AI.^[125]^ This task necessitates a user-centered design with clinician input. Besides, the cost–benefit ratio of deploying and maintaining AI systems must be carefully evaluated with respect to their intended applications.^[126,127]^

As AI evolves, clinicians will require training to leverage these technologies while understanding their limitations,^[128]^ and clear guidelines should be established on the correct use of AI in practice. Ultimately, AI should not replace clinicians but rather work synergistically with them.^[129]^

Research is ongoing to address these limitations through advances in data science, human–AI interaction, regulations, and education of both clinicians and the general public. With continued progress, it appears that AI can enable more efficient, precise, and equitable eye care globally.

### Perspective

The outlook for AI in retinal disease diagnosis and management looks promising. Recent developments in DL methods have prompted significant progress in automated retinal image analysis, hence enabling the detection of a wide range of pathological conditions. Notwithstanding these advancements, the field continues to confront impediments that necessitate additional exploration and refinement.

One key area is expanding the capabilities of AI systems beyond binary disease classification to provide a more granular characterization of disease severity, progression, and prognosis. As demonstrated by Maunz et al, AI models can potentially predict visual acuity outcomes in neovascular AMD based on quantitative analysis of OCT biomarkers.^[112]^ Further research is needed to develop similar prognostic capabilities for other retinal conditions.

There is also a need to validate AI systems prospectively through clinical trials before their widespread deployment in real-world screening and diagnostics. Following FDA clearance of the IDx-DR system, a more rigorous evaluation of AI tools will be crucial to gaining physician trust and adoption.^[130]^

Finally, interpretability remains a key challenge. While AI models can match or exceed human performance on well-defined tasks, understanding how they arrive at predictions is limited. Building hybrid systems that synergistically combine computer and clinician capabilities could maximize the strengths of both.^[131]^

In short, AI promises to transform retinal disease management but there is a need for ongoing research and validation to realize its full potential. It appears that multi-disciplinary teams tackling these open problems will shape the next phase of AI integration in ophthalmic care.

##  Summary

AI has demonstrated tremendous potential to transform ophthalmology through its applications in screening, diagnosis, treatment planning, and improving clinical workflows. DL has provided unique opportunities in the interpretation of ophthalmological data, including retinal imagery, OCT scans, visual field assessments, and the identification of various ocular diseases. AI systems have reached a diagnostic precision comparable to, or even surpassing, that of healthcare professionals in identifying common eye diseases such as DR, AMD, and glaucoma.

However, there remain challenges to translate these research applications into widespread clinical practice. The lack of large, diverse, high-quality training datasets limits generalizability across patient populations and settings. Most AI systems still lack regulatory approval and validation through multicenter clinical trials. There are concerns also about legal implications and clinician trust in "black box" algorithms, and it is vital to properly integrate them into existing workflows while addressing costs and infrastructure requirements.

These limitations should be closely addressed through robust research, explainable AI models, pragmatic clinical validation studies, and stakeholder engagement so that AI is enabled to contribute to earlier disease detection, optimal resource allocation, personalized treatments, and improved patient outcomes. AI-empowered intelligent screening programs could provide equitable access and exponential gains in efficiency. Furthermore, collaborative human–AI systems that combine the strengths of clinicians and technology could set a new standard for evidence-based, precise care.

### Financial Support and Sponsorship 

None.

### Conflicts of Statement

None.
